# Dual mechanisms regulating *glutamate decarboxylases* and accumulation of gamma-aminobutyric acid in tea (*Camellia sinensis*) leaves exposed to multiple stresses

**DOI:** 10.1038/srep23685

**Published:** 2016-03-29

**Authors:** Xin Mei, Yiyong Chen, Lingyun Zhang, Xiumin Fu, Qing Wei, Don Grierson, Ying Zhou, Yahui Huang, Fang Dong, Ziyin Yang

**Affiliations:** 1 Key Laboratory of South China Agricultural Plant Molecular Analysis and Genetic Improvement & Guangdong Provincial Key Laboratory of Applied Botany, South China Botanical Garden, Chinese Academy of Sciences, Xingke Road 723, Tianhe District, Guangzhou 510650, China; 2 University of Chinese Academy of Sciences, No.19A Yuquan Road, Beijing 100049, China; 3 College of Horticultural Science, South China Agricultural University, Wushan Road, Tianhe District, Guangzhou 510642, China; 4 Plant & Crop Sciences Division, School of Biosciences, University of Nottingham, Sutton Bonington Campus, Loughborough LE12 5RD, UK; 5 Guangdong Food and Drug Vocational College, Longdongbei Road 321, Tianhe District, Guangzhou 510520, China

## Abstract

γ-Aminobutyric acid (GABA) is one of the major inhibitory neurotransmitters in the central nervous system. It has multiple positive effects on mammalian physiology and is an important bioactive component of tea (*Camellia sinensis*). GABA generally occurs at a very low level in plants but GABA content increases substantially after exposure to a range of stresses, especially oxygen-deficiency. During processing of tea leaves, a combination of anoxic stress and mechanical damage are essential for the high accumulation of GABA. This is believed to be initiated by a change in glutamate decarboxylase activity, but the underlying mechanisms are unclear. In the present study we characterized factors regulating the expression and activity of three tea *glutamate decarboxylase* genes (*CsGAD1, 2*, and *3*), and their encoded enzymes. The results suggests that, unlike the model plant *Arabidopsis thaliana,* there are dual mechanisms regulating the accumulation of GABA in tea leaves exposed to multiple stresses, including activation of CsGAD1 enzymatic activity by calmodulin upon the onset of the stress and accumulation of high levels of *CsGAD2* mRNA induced by a combination of anoxic stress and mechanical damage.

γ-Aminobutyric acid (GABA) is a non-proteinaceous amino acid that occurs in animals, plants, and bacteria. In animals, GABA is known to function as one of the major inhibitory neurotransmitters in the central nervous system^1^, and may have multiple positive effects on reducing hypertension and regulating blood pressure^2^,^3^,^4^,^5^, reducing anxiety^6^, modulating renal function^7^, and inhibiting metastasis of cancer cells^8^. In plants, GABA is proposed to be involved in pH regulation^9^, signaling^10^,^11^, redox regulation^12^, stress responses^13^, regulation of carbon and nitrogen metabolism/ energy balance, osmoregulation, plant growth, and defense against insects^14^,^15^,^16^.

The potent bioactivity of GABA has led to intensive studies on the genes and enzymes involved in its biosynthesis. GABA is synthesized via a GABA shunt pathway involving the direct and irreversible α-decarboxylation of glutamate to GABA by glutamate decarboxylase (GAD), followed by the reversible conversion of GABA to succinic semialdehyde by GABA transaminase (GABA-T), and the irreversible oxidization of succinic semialdehyde (SSA) to succinate by SSA dehydrogenase (SSADH), which can be catabolized to γ-hydroxybutyrate (GHB) by succinic semialdehyde reductase (SSR)/ glyoxylate reductase (GLYR)^15^,^17^,^18^. GAD is the most extensively characterized of the GABA pathway enzymes and has been studied in many plants such as *Arabidopsis*^19^, tobacco^20^, tomato^21^, petunia^22^, soybean^23^, and *Panax ginseng*^24^.

GABA normally occurs in plants at a relative low level but the GABA content increases by up to a dozen-fold after exposure to a range of stresses including mechanical damage, acidosis, salinity, heat, cold, drought, virus infection, and low-oxygen^13^,^25^,^26^. Oxygen-deficiency stress generally has a remarkable influence on the accumulation of GABA and investigations in *Arabidopsis thaliana* suggest that GABA increases in response to low oxygen caused by treatment with argon gas, submergence, and water logging, due to the activation of GAD enzymatic activity rather than enhanced mRNA transcription or translation^27^,^28^. Cytosolic acidification, promoted by hypoxia and anoxia, activates GAD^29^,^30^ and many plant GADs are reported to be activated by Ca^2+^/calmodulin (CaM), especially under stress conditions such as hypoxia, where cytosolic calcium increases at the onset of the stress^19^,^22^,^28^,^31^,^32^. The putative CaM-binding domain is conserved among the CaM/Ca^2+^-dependent GADs, in *Arabidopsis*^19^, tobacco^20^, tomato^21^, petunia^22^, soybean^23^, and *Panax ginseng*^24^ and contains two positively charged segments (Lys474-Lys475 and Arg495-Lys496-Lys497) and a conserved Trp-Lys-Lys-Phe-Val motif^33^. However, in the rice GAD *OsGAD2,* these three motifs are incomplete or lacking and the protein is unable to bind CaM^34^,^35^ and two *A. thaliana* GADs, AtGADs 3 and 5, and four GADs from soybean and one from maize have also been suggested to be unable to bind CaM^33^. In addition, one apple (*Malus* x *domestica* Borkh.) MdGAD3 was not regulated by calmodulin or auto-inhibited by the C terminus^36^.

Tea (*Camellia sinensis*) is an important crop in around 30 countries including China, Japan, India, Kenya^37^ and is the most widely consumed beverage aside from water. High accumulation of GABA under anoxic conditions occurs in picked tea leaves as in other plants^13^,^38^, although the reason why anoxic stress can induce the accumulation of GABA in tea leaves is still unknown. The manufacture of the familiar GABA tea product involves an anoxic-stress, unlike the *Arabidopsis* experiments cited above, but also involves mechanical damage (ie picking and handling). Several key questions remain to be answered. (1) What are the potential roles of calmodulin activation and GAD transcript levels in determining the elevated levels of GABA in tea leaves exposed to anoxia and mechanical stress? (2) Which specific GAD isoform is involved in GABA production in tea leaves? (3) Do any other stresses, in addition to anoxic stress, such as mechanical stress associated with picking and handling, play a role in the formation of GABA in plants?

We successfully identified several enzymes involved in the formation of tea aroma by expression in *E. coli* and transient expression in the model plant *N. benthamiana*^39^,^40^,^41^ and in the present study we isolated three *GADs* (*CsGAD1, 2*, and *3*) from tea leaves and demonstrated they encoded enzymes capable of converting glutamate to GABA. Studies on their properties and expression during tea processing showed that stress response patterns and mechanisms related to GABA production in tea leaves are more complex than in the model plant *Arabidopsis thaliana* and help to explain the large increase in GABA during tea processing.

## Results and Discussion

### Loss of glutamate and concomitant accumulation of GABA in tea leaves in response to anoxic stress and mechanical damage

**Figure 1:**
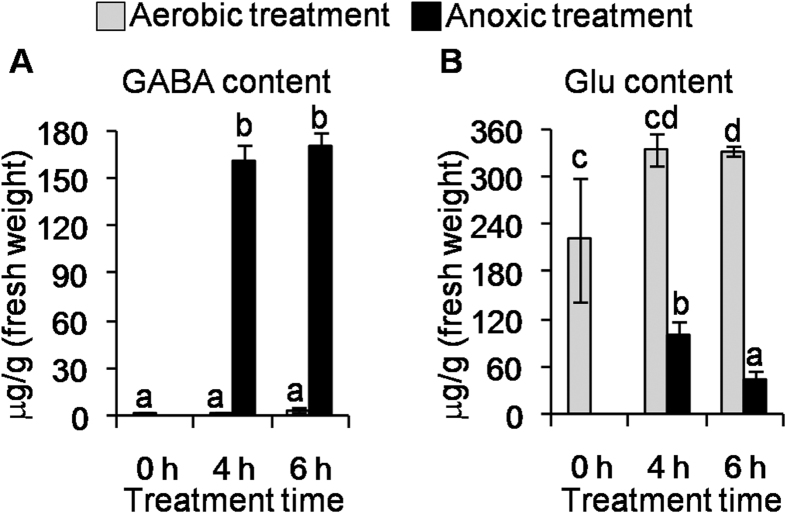
Contents of GABA (**A**) and Glu (**B**) of picked, mechanically damaged, tea (*C. sinensis* cv. Jinxuan) leaves under aerobic and anoxic treatments. GABA, gamma-aminobutyric acid. Glu, glutamate. Data are expressed as mean ± S.D. (n = 3). Different means with different letters are significantly different from each other (*p* ≤ 0.05).

The GABA content in tea (*C. sinensis* cv. Jinxuan) leaves, which had already suffered mechanical damage due to picking and handling, increased significantly under anoxia in contrast to the aerobic control (Fig. 1A) and there was a concomitant decrease in the precursor glutamate (Fig. 1B and Table S1). This suggested that the pathway involving glutamate to GABA may play a key role in accumulation of GABA under anoxia. The accumulation of GABA under anoxic stress is observed in many plants including most cultivars of picked tea leaves^38^,^42^,^43^ but it is important to emphasise the mechanical damage these leaves experience as a result of picking and handling (Figure S1). Besides GABA and glutamate, we also found the content of some other amino acids such as aspartic acid, alanine, asparagine, glycine, citrulline, and histidine were affected by anoxia (Table S1).

### CsGAD1 and CsGAD2 convert glutamate to GABA in *E. coli* and in plants

**Figure 2:**
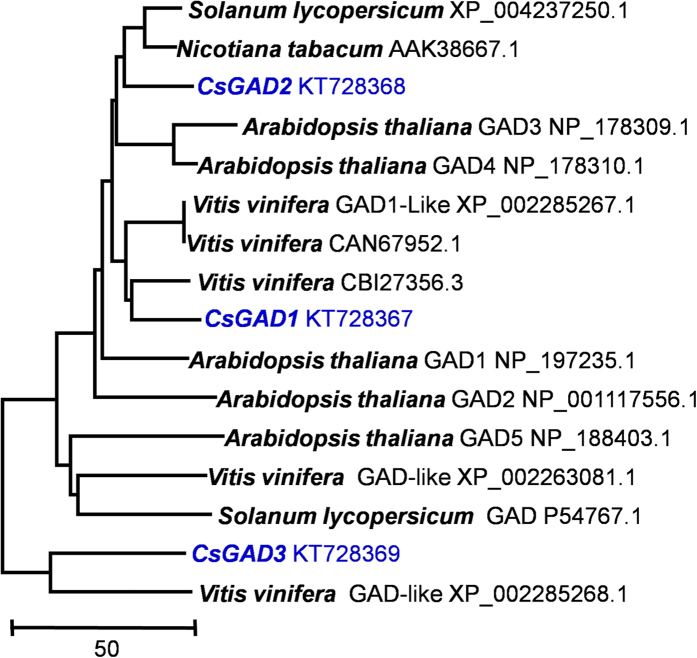
Phylogenetic analysis of CsGADs and related functionally characterized GADs. GAD, glutamate decarboxylase. Sequences were aligned using the ClustalX program. The neighbor-joining phylogenetic tree was generated by MEGA 5 software. The proteins included in the tree are represented by GenBank accession number.

Since the metabolic profiling indicated that GAD may play a key role in accumulation of GABA under anoxia, we identified GADs expressed in tea leaves by searching the *C. sinensis* Transcriptome Shotgun Assembly (TSA) database of NCBI. By blasting and splicing different mRNA sequences, three GAD ORFs were found and designated CsGADs and using primers (Table S2) three CsGADs from tea (*C. sinensis* cv. Jinxuan) leaves were isolated and sequenced (CsGAD1 GenBank Accession KT728367; CsGAD2 GenBank Accession KT728368; CsGAD3 GenBank Accession KT728369). CsGAD1 and CsGAD2 have ORFs of 1482 nucleotides encoding polypeptides of 493 amino acids, with molecular weights of 55.5 and 55.7 kDa respectively, and the 1539 nucleotides CsGAD3 ORF encodes a polypeptide of 512 amino acids with a calculated protein molecular mass of 58.0 kDa. Phylogenetic analysis performed using a Clustal method (Fig. 2) indicated that the amino acid sequence of CsGAD2 showed high homology with sequences from tomato and tobacco, while CsGAD1 and CsGAD3 were closely related to two grape GADs (Fig. 2).

**Figure 3:**
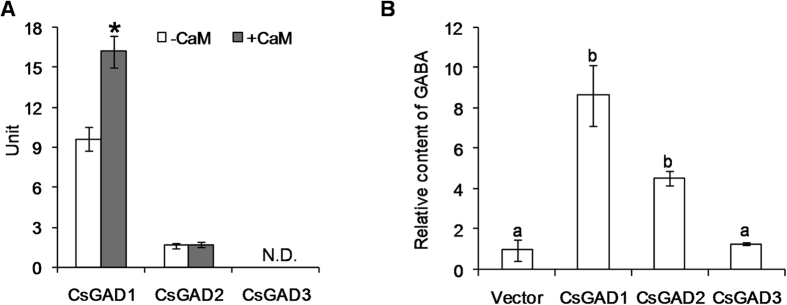
Analysis of recombinant CsGADs enzyme activities in *E. coli* (**A**) and overexpressed in *N. benthamiana* (**B**). (**A**) One unit of enzyme activity was defined as the amount (μmol) of GABA produced by the action of one mg protein per min. The white columns indicate the activities of CsGADs recombined in *E. coli*. The grey columns indicate the activities of the CsGADs in the presence of CaM. *shows the significant difference (*p* < 0.05) of CsGAD1 activity in the absence and presence of CaM. N.D. indicates that the CsGAD3 had no activity converting glutamate to GABA. (**B**) Vector, empty vector group as control. CsGAD1, CsGAD2, CsGAD3: overexpression of CsGAD1, CsGAD2, and CsGAD3 respectively. The GABA content in the Vector group was defined as 1. Data represent the mean value ± standard deviation of three independent experiments performed in triplicate. Different means with different letters are significantly different from each other (*p* ≤ 0.05). GAD, glutamate decarboxylase.

To date it is difficult to characterize the functions of enzymes involved in biosynthesis of plant metabolites such as tea, where there is no genetic transformation system, and functional characterization of these tea CsGADs was conducted in an *E. coli* system and following transient expression in *N. benthamiana* plants. Figure S2 shows the SDS-PAGE and Western Blot analyses of CsGADs expressed in *E.coli*. The recombinant CsGAD1 and CsGAD2 were able to convert glutamate to GABA when expressed in *E. coli*, whereas the recombinant CsGAD3 had no activity (Fig. 3A). Furthermore, the transient expression of *CsGAD1* and *CsGAD2* (Figure S3) in *N. benthamiana* plants led to higher GABA contents in these plants compared to those treated with empty vector as control (Fig. 3B). Results from both expression systems were consistent and established that *CsGAD1* and *CsGAD2* could catalyze the conversion of glutamate to GABA in plants. Previously, GAD enzyme activity has only been reported in a crude extract of tea leaves^42^ and our study identified two functional GAD enzymes for the first time.

### CsGAD1 enzyme activity can be activated by binding CaM

**Figure 4:**
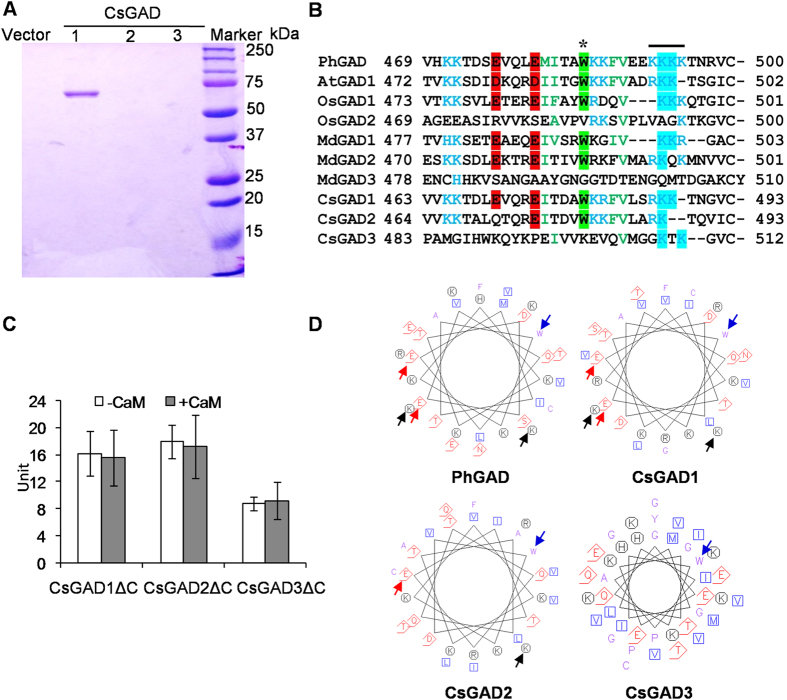
Analysis of CaM binding ability of recombinant CsGADs expressed in *E. coli* (**A**) C-terminal truncated regions of the CsGADs (**B**) enzyme activities of CsGADΔCs (**C**), and comparison of properties of amino acids in the CaM binding domains of GADs (D). GAD, glutamate decarboxylase. GADΔC, C-terminal truncated glutamate decarboxylase. (**A**) SDS-PAGE of proteins retained by CaM-affinity chromatography. Only CsGAD1 was visible following PAGE, suggesting that only CsGAD1 had CaM-binding ability. (**B**) The clusters of Trp (W) and Lys (K) present in the C-proximal regions of GADs, which are important for *in vitro* binding of CaM, are indicated by asterisks and a thick line, respectively. The positions of two pseudosubstrate residues are highlighted in red. Based on comparison with the *PhGAD* (L16977), *AtGAD1* (At5g17330), *OsGAD1* (AB056060), *OsGAD2* (AB056061), *MdGAD1* (KC812242), *MdGAD2* (KC812243), and *MdGAD3* (KC812244), these amino acids of CsGADs were truncated to express CsGADΔCs in *E. coli*. (**C**) One unit was defined as the amount (μmol) of GABA produced by the action of one mg protein in min. The white columns indicate the activities of recombinant CsGADΔCs in *E. coli*. The grey columns indicate the activities of the CsGADΔCs in the presence of CaM. (**D**) The α-helix was presented using the EMBOSS-Lite-Protein Analysis Tools. The Trp (W) -centered (blue arrows) hydrophobic residues are clustered on one side, with the hydrophilic region on the other side. The Trp (W) residue and the Lys (K) (black arrows) cluster, contribute to hydrophobic and electrostatic interactions and are critical for efficient binding of CaM to the PhGAD-CaM binding domain^22^,^31^. The Glu (**E**) (red arrows) residues function as pseudosubstrates.

The question whether CsGAD1 and CsGAD2 enzymes could bind and be activated by CaM was investigated. Only CsGAD1 could be activated by CaM (Fig. 3A) and, moreover, CaM-affinity chromatography revealed that only CsGAD1 was capable of binding CaM (Fig. 4A).

The C-terminal regions of CsGADs contain a putative CaM-binding domain and C-terminal truncated CsGADΔCs were constructed and cloned (Fig. 4B and Figure S4) in order to investigate the role of the C-termini in CaM activation. None of the CsGADΔCs, including CsGAD1ΔC, was activated by CaM (Fig. 4C). Moreover, the enzyme activities of CsGADΔCs (Fig. 4C) were higher in the absence of CaM than those of the full length CsGADs (Fig. 3A). CsGAD3ΔC, in particular, had a catalytic activity converting glutamate to GABA when the C-proximal region was absent. None of the CsGADΔCs was selected by CaM-affinity chromatography (Figure S5), suggesting that they lacked the ability to bind CaM. Thus, the C-terminal regions of CsGADs (Fig. 4B) appear to be involved in regulating both CaM-binding and the enzyme catalytic activity.

Many studies have validated the important role of GAD in the accumulation of GABA under low-oxygen. It is generally accepted that anoxia stress does not directly affect GAD activity but increases cytosolic Ca^2+^, which complexes with CaM^44^. Many plant GAD enzymes are reported to be activated by binding of CaM to the C-terminal regions of GADs^19^,^22^,^28^,^31^,^32^. The amino acid sequences of the C-terminal regions of CsGADs were compared with those from *Arabidopsis thaliana* AtGADs, *Oryza sativa* OsGADs, and *Petunia hybrida* PhGAD (Fig. 4B). The overall similarity among the C-terminal extensions of these plant GADs is not high; however, CsGAD1, which can bind CaM (Fig. 4A) and be activated by CaM (Fig. 3A), had a similar CaM-binding domain (Fig. 4B) to other CaM-dependent GADs including PhGAD^22^, AtGAD1^19^, OsGAD1^35^, and MdGAD1^45^ and several amino acids in these CaM-dependent GADs are conserved (Fig. 4B). The α-helix analysis of PhGAD indicated that all hydrophobic residues are clustered on one side, with positive charges on the opposing side (Fig. 4D). In particular, the Trp (W) residue and the Lys (K) cluster, which contribute to hydrophobic and electrostatic interactions respectively, are critical for efficient binding of CaM to the PhGAD-CaM binding domain^31^. CsGAD1 was identical to PhGAD at these important sites, whereas CsGAD2 lacked one Lys (K) and one Glu (E) and CsGAD3 had a quite different structure (Fig. 4D). In the present study, the CaM binding domain of CsGAD1 was only demonstrated *in vitro*. It remains to be determined if CsGAD1 is regulated by calmodulin *in vivo*. Further experiments such as isolation of the mesophyll cells and investigation of the response *in vivo* conducted by Cholewa *et al*.^46^ are required to provide more definitive evidence about *in vivo* function.

The two lysines, K496 and K497, serve as anchoring sites for CaM binding in the AtGAD1-CaM complex. In the mutational analysis, removing either or both caused the loss of the ability to bind Ca^2+^/CaM^45^. Similarly, PhGAD, OsGAD1, MdGAD1, and CsGAD1, which have K496 and K497, can all bind CaM, whereas OsGAD2, MdGAD3, CsGAD2, and CsGAD3 lack K496 and K497 and cannot bind and are not activated by CaM^35^,^36^ (Figs 3 and 4). In addition, pseudosubstrate Glu residues (E476 and E480) in the C-terminal regions of GADs have frequently been observed in plant GADs, except OsGAD2 and MdGAD3^47^. In the present study, CsGAD2 and CsGAD3 were found be the exceptions to this generalization (Fig. 4B). Interestingly, when the C-terminal regions of CsGADs were truncated, the CsGADΔCs exhibited higher activities than CsGADs (Figs 3 and 4). Taken together, these observations suggest that (1) the C-terminal extensions of CsGADs contain sequences capable of inhibiting enzyme activity; (2) the K496 and K497 may be key sites for CaM binding in the CsGAD-CaM complex, causing inhibition.

### Enhanced accumulation of *CsGAD2* mRNA contributes to accumulation of GABA in tea leaves under combined anoxia stress and mechanical damage

**Figure 5:**
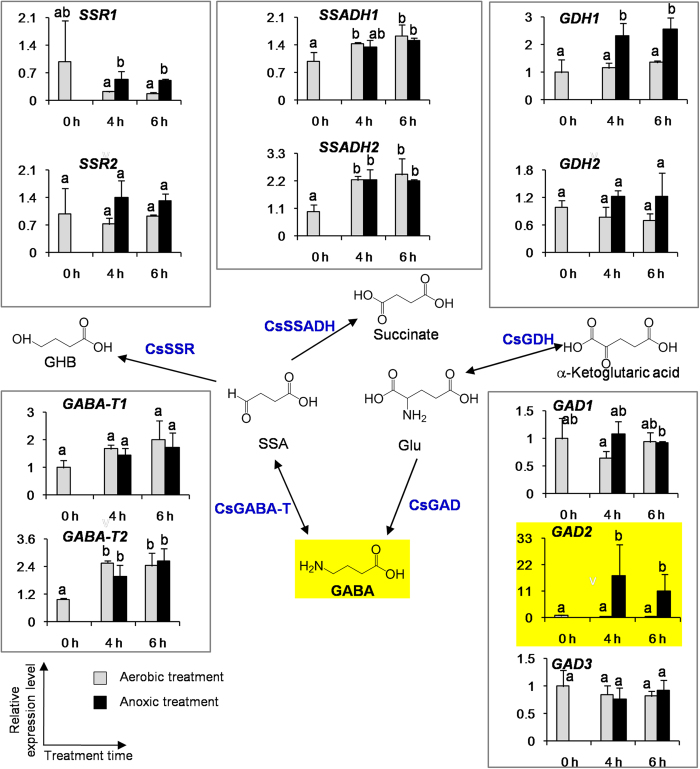
Effect of anoxic treatment on the transcript levels of the genes involved in formation and metabolism of GABA in the picked tea leaves. GABA-T, GABA transaminase. GAD, glutamate decarboxylase. GDH, glutamate dehydrogenase. SSADH, succinic semialdehyde dehydrogenase. SSR, succinic semialdehyde reductase. The tea leaves were from *C. sinensis* cv. Jinxuan. Data are expressed as mean ± S.E. (n = 3). Transcript abundance was calculated based on the difference in cycle threshold (Ct) values between the target gene and beta-actin transcripts normalized by the 2^−△△Ct^ relative quantification method. The mRNA levels of the genes in the picked tea leaves at 0 h were defined as 1. Different means with different letters are significantly different from each other (*p* ≤ 0.05).

To investigate if the expression levels of genes involved in formation of GABA were affected by anoxic stress and mechanical damage, we looked at *CsGADs, CsGABA-Ts, CsSSRs, CsSSADHs*, and *CsGDHs* (*GDH, glutamate dehydrogenase*). Only *CsGAD2* was highly expressed in picked tea leaves subjected to anoxic treatment, in contrast to the aerobic treatment (Fig. 5). Investigation of several cultivars of tea leaves gave a similar result ie only *CsGAD2* mRNA accumulates in picked tea leaves under anoxic treatment in contrast to the aerobic treatment (Figure S6), which is consistent with the increment in GABA content in these tea cultivars (Figure S1). These observations suggest that, in addition to CaM activation, enhanced accumulation and/or translation specifically of *CsGAD2* mRNA may also play an important role in the accumulation of GABA in tea leaves exposed to combined anoxic stress and mechanical damage.

We investigated changes in GABA content and *CsGADs* mRNA levels in intact tea leaves exposed to anoxia to see whether they were similar to those in picked leaves exposed to the same conditions. In the intact tea leaves, GABA content was increased 3.5 fold under anoxic treatment in contrast to aerobic treatment (Figure S7A), 23-fold lower than the 80-fold increase observed in postharvest (picked) tea leaves under anoxic treatment (Fig. 1). Significantly, the levels of *CsGADs* mRNAs in the intact tea leaves were not affected by the anoxic treatment alone (Figure S7B), confirming the important role of mechanical damage from picking in enhancing *CsGAD2* mRNA levels in accumulation of GABA, through enhanced transcription or translation or both, in tea leaves exposed to anoxia.

In response to other environmental stresses except anoxia and mechanical damage, GABA production often increases so much that cellular levels of this non-protein amino acid exceed that of amino acids involved in protein synthesis. This situation has been reported in drought-stressed cotton, bean leaves, and turnip leaves, viral or salt-stressed tomato leaves, cold-stressed soybean leaves and asparagus cells, acidosis-stressed asparagus cells, and heat-stressed cowpea cells (Reviewed in ref. 13). Experimental evidence supports the involvement of GABA synthesis in pH regulation, nitrogen storage, plant development and plant defence against insect attack, as well as a compatible osmolyte and an alternative pathway for glutamate utilization^13^,^15^. Environmental stresses increase GABA accumulation through two different mechanisms^13^,^15^. Firstly, stresses causing metabolic and/or mechanical disruptions lead to cytosolic acidification, which induces an acidic pH-dependent activation of GAD and GABA synthesis. Secondly, these stresses initiate a signal-transduction pathway, in which increased cytosolic Ca^2+^ stimulates Ca^2+^/calmodulin-dependent GAD activity and GABA synthesis. Apart from activation of GADs by CaM, there is scant information available about the regulation of genes or enzymes involved in GABA formation in response to anoxic stress. In *A. thaliana*, expression of the genes involved in the GABA shunt showed little response to anoxia, except for *AtGAD3/4*, which has closest homology to *CsGAD2*, and increased slightly^28^. In the previous reports on GABA content of plants in response to anoxia, the history of the leaves (whether intact, picked, or handled) was not clearly described. Leaves from tea plants experience mechanical damage during picking and handling, in contrast to leaves of intact plants. This means that in some reports there may have been two stresses involved in the formation of GABA. In the present study, anoxic stress had a much greater effect on the GABA contents and expression levels of *CsGADs* in picked tea leaves compared to intact tea leaves, suggesting that mechanical damage stress from the picked leaves is essential for the formation of high levels of GABA in response to the anoxia. In the model plant *A. thaliana* we showed (Figure S8) that (1) GABA content was not increased either in intact or in picked leaves in response to the anoxic stress, but was increased in the picked leaves compared with the intact leaves. (2) Expression levels of *AtGADs* in the picked leaves were much higher than those of the intact leaves (note the difference in scales in Figures S8A and S8B). This suggests that GABA content and expression levels of *AtGADs* were more dependent on the mechanical damage stress than the anoxic stress. In addition, single stresses differentially influenced *AtGADs* expressions, especially *AtGAD4*, in *Arabidopsis*.

**Figure 6:**
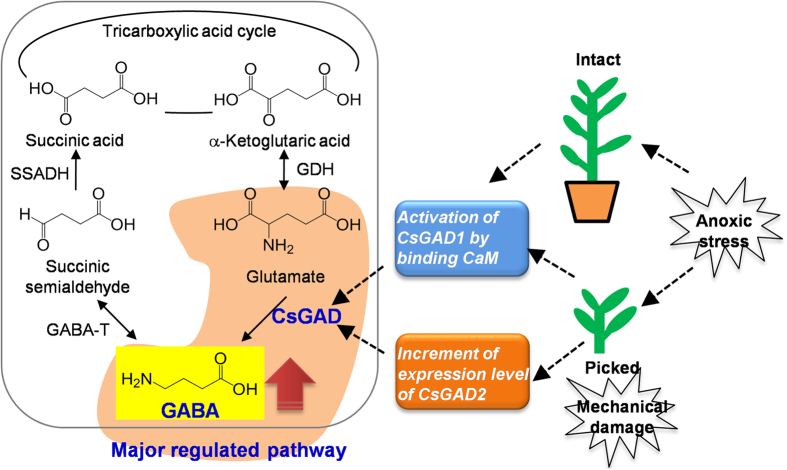
Hypothetical model of GABA formation in intact and picked tea leaves exposed to anoxic stress. GABA, gamma-aminobutyric acid. GABA-T, GABA transaminase. GAD, glutamate decarboxylase. GDH, glutamate dehydrogenase. SSADH, succinic semialdehyde dehydrogenase. SSR, succinic semialdehyde reductase.

In conclusion, this study for the first time provides evidence that accumulation of GABA in tea leaves under combined anoxic stress and mechanical damage is associated with both enhanced mRNA accumulation and CaM-activated enzymatic activity of CsGADs. CsGAD1 has a calmodulin-regulated autoinhibitory domain, whereas CsGAD2 is probably transcriptionally regulated. Both CsGAD2 and CsGAD3 have an autoinhibitory domain that is independent of calmodulin. Moreover, picked tea leaves and intact tea leaves show different responses to anoxic stress (Fig. 6). The combination of stress due to mechanical damage and anoxia are essential for the dramatic accumulation of GABA, which is quite different from the stress responses of the model plant *A. thaliana*. This information will advance our understanding of molecular mechanisms of GABA accumulation in plants, and provides essential information for the development of GABA tea products.

## Methods

### Plant materials and treatments

The leaves of *C. sinensis* cv. “Jinxuan”, “Huanong No. 2”, “Fuyun No. 6”, “Renhuabaihao”, and “Yinghong No. 9”, which are popular tea cultivars in South China, were obtained from the Tea Experiment Station in the South China Agricultural University (Guangzhou, China) in June, 2013 and 2014. For the comparison between intact tea leaves (without mechanical damage) and picked tea leaves (with mechanical damage) in response to anoxia, we used only *C. sinensis* cv. “Jinxuan” leaves obtained from seedlings grown under controlled conditions of 22–25 °C and 12 h light/ 12 h dark. The anoxic treatments were carried out using a Vacuum Sealer (Deni, TVS-2013) at 25 °C, 70% humidity in the dark. The mechanical damage was induced by picking. There were two anoxic treatments (Figure S9, Supplementary information), including (1) anoxic treatment of picked tea leaves (a combination of anoxic stress and mechanical damage); (2) anoxic treatment of intact tea leaves (as a single anoxic stress). After 4 h or 6 h treatment, one bud and three leaves were ground in liquid nitrogen and stored at −80 °C until use. In addition, *Arabidopsis thaliana* (Col-0) exposed to single anoxic stress, and anoxic stress plus mechanical damage together were also prepared using the same treatments as for tea leaves.

### Analysis of free amino acids

The extraction protocol of free amino acids was described by Yang *et al*.^48^. Plant tissues (500 mg fresh weight, finely powdered) were extracted with 1.5 ml cold methanol (100%) by vortexing for 2 min followed by ultrasonic extraction in ice cold water for 15 min. The extracts were mixed with 1.5 ml chloroform and 0.6 ml cold water for phase separation. The resulting upper layer was dried and used as the crude extract of amino acids.

For analysis of all the free amino acids, the dried crude extract was dissolved in 200 μl of 5% sulfosalicylic acid, and stood for 1 h. After centrifuging at 12000 × *g* for 10 min, the supernatant was filtered through a 0.45 μm membrane, and subjected to a Hitachi L-8800 amino acid analyzer (Tokyo, Japan). The experiments were performed on a high efficiency sodium cation-exchange Hitachi 855-350 column (4.0 × 150 mm). The system was operated using a mobile phase consisting of lithium citrate pH 2.9, pH 4.2, pH 8.0, and using UV-Vis detection at 570 nm and 440 nm. The flow-rate of the mobile phase was 0.45 ml/min, and the flow-rate of the derivatizating reagent was 0.25 ml/min. The column temperature was set at 38 °C, and the post column reaction equipment was maintained at 130 °C. The temperature of the auto-sampler was kept at 5 °C, and the injection volume was 50 μl for both standard and samples. The areas under the peaks corresponding to the amounts of amino acids were compared to the amino acid standards.

For analysis of GABA, the dried crude extract was dissolved in 100 μl of pyridine, and derivatized with 50 μl of *N*-methyl-*N*-(trimethylsilyl) trifluoroacetamide (MSTFA) at 37 °C for 60 min, cooled, and then centrifuged. The MSTFA derivates were then analyzed by a gas chromatograph-mass spectrometer QP2010 SE (Shimadzu Corporation, Japan). The injector temperature was 240 °C, splitless mode was used with a splitless time of 1 min, and helium was the carrier gas with a velocity of 1.0 ml/min. An HP-5 column (Agilent Technologies, 30 m × 0.25 mm × 0.25 μm) was used with an initial temperature of 100 °C for 2 min, a ramp of 5 °C/min to 300 °C, and then a hold at 300 °C for 10 min^49^. The mass spectrometer was operated with full scan mode and SIM mode (*m/z* 174 and 304 for GABA-MSTFA derivate). The content of GABA in samples was calculated based on linear formula of different concentrations of authentic GABA standard.

### Gene cloning and sequence analysis of CsGADs and their C-termini-truncated CsGADΔCs

Three GAD enzymes (CsGAD1, 2 and 3) were selected from a tea transcriptome database. The C-proximal regions of CsGADs were truncated (detailed information is shown in the Results section). Total RNA was isolated from finely powdered plant tissues (100 mg) using a Spectrum Plant Total RNA Kit (Sigma-Aldrich, St. Louis, CA, USA). The first strand cDNA was synthesized using the Reverse Transcription System (Promega, Madison, WI, USA). The full length open reading frames (ORF) were amplified from first strand cDNA with primers listed in Table S2. The resulting PCR products were purified and subcloned into the pGEM-T vector (Promega, Madison, WI, USA) and sequenced. Sequences were aligned using the ClustalX program. The phylogenetic tree was calculated by neighbor-joining algorithms and generated by MEGA 5 software^50^. Analysis of the CaM-binding domain sequences utilized the high-performance computational capabilities of the Helix Systems at the National Institutes of Health, Bethesda, MD (http://helix.nih.gov).

### Transcript expression analysis

Total RNA was isolated immediately after dissection using a Quick RNA isolation Kit (Huayueyang Biotechnology (Beijing) Co., LTD., Beijing, China). The reactions were performed using iTaq^TM^ Universal SYBR^®^ Green Supermix (BioRad, Hercules, CA, USA) in a 20 μl volume containing 10 μl of iTaq^TM^ Universal SYBR^®^ Green Supermix (2 × ), 0.4 μM each specific forward and reverse primer. 2 μl of 10-fold diluted template was used for 50 μl PCR reaction using primers listed in Table S2. The qRT-PCR was carried out in a Roche LightCycle 480 (Roche Applied Science, Mannheim, Germany) with one cycle of 95 °C 30 s, 40 cycles of 95 °C 5 s, 60 °C 30 s. A melt curve was performed at the end of each reaction to verify PCR product specificity. The 2^−△△Ct^ method was used to calculate the relative expression level^51^. Beta-actin was used as an internal refs 39, 40, 41. Changes in mRNA level of the test gene for each treatment were normalized to that of beta-actin.

### CsGADs and CsGADΔCs expression in Escherichia coli

The full-length ORFs of Cs*GADs* and the C-termini truncated *CsGADΔCs* were subcloned into pET-32a vector (Novagen, Madison, WI, USA) to obtain the expression construct. After verification by sequencing, the expression construct was transformed into *Escherichia coli* Rosetta (Novagen, Madison, WI, USA) for inducible His-tagged and S-tagged protein expression. Freshly transformed Rosetta cells harboring pET-32a/GADs or empty pET-32a vector were grown at 37 °C to an O.D.600 = 0.6. After adding 0.1 mM isopropyl-beta-D-thiogalactopyranoside (Sigma-Aldrich, St. Louis, CA, USA), the cultures were grown at 20 °C for another 10 hours to produce recombinant His-tagged and S-tagged protein. The cells were harvested at 4000 × *g* for 10 min, then disrupted by sonication for 30 min (sonication 0.5 s at 1% strength and wait for 1.5 s; instrument: JY92-IIDN, Ningbo, China) in a 50 mM NaH_2_PO_4_ (pH 8.0) buffer containing 300 mM NaCl and 10 mM imidazole. After centrifugation at 10000 × *g* for 20 min, the supernatant was collected and purified by using affinity binding on Ni-NTA resin (Qiagen, Chatsworth, CA, USA) according to the manufacturer’s instruction. The purified protein was passed through a PD-10 desalting column and the His-tag and S-tag was removed by enterokinase digestion (Novagen, Madison, WI, USA).

To further determine if the expressed proteins had CaM-binding ability, they were filtered through a CaM-Sepharose column and the expressed protein supernatant was loaded onto Calmodulin sepharose 4B (1 ml, GE Healthcare), which was pre-equilibrated with 50 mM Tris-HCl buffer (pH 7.5) containing 150 mM NaCl and 1 mM CaCl_2_. Afterwards, the column was washed with 20 bed volumes of 25 mM Tris-HCl buffer containing 150 mM NaCl. Finally, 1 ml of 50 mM Tris-HCl (pH 7.5) containing 2 mM EDTA and 150 mM NaCl was applied to elute the column to obtain any CaM-binding proteins which were fractionated by SDS-PAGE.

### Western blot analysis

The expressed proteins in *E. coli* were resolved on a 10% SDS-polyacrylamide gel and transferred to a polyvinylidene fluoride membrane, which was then probed with an anti-His × 6 antibody (Signalway antibody, Pearland, TX, USA) at a dilution of 1: 3000, followed by an anti-mouse secondary antibody (Signalway antibody, Pearland, TX, USA) at a dilution of 1: 10000. The S-tagged proteins were detected with a SuperSignal West Pico Chemiluminescent Substrate (Pierce, Rockford, IL, USA) according to the manufacturer’s instruction.

### CsGAD, CsGADΔCs and their binding CaM enzyme assay

The 400 μl (0.04 ~ 4 μg) of GAD or CsGADΔCs enzyme reaction system contained 200 mM pyridine-HCl (pH 5.8), 3 mM glutamate, 20 μM phosphopyridoxal, and the purified recombinant protein. For the determination of CaM-binding ability of CsGAD or CsGADΔCs, 0.3 mM CaCl_2_ and 0.4 mM bovine CaM (Sigma) were added to the above reaction system. The reaction was conducted at 30 °C for 60 min and terminated by addition of 1/10 volume of 1 N HCl. 200 ng of ribitol was added as an internal standard, the resultant solution was freeze-dried, dissolved in 100 μl of pyridine, derivatized with 50 μl of MSTFA at 37 °C for 60 min, cooled, and then centrifuged and the GABA-MSTFA derivates were then analyzed by GC-MS as described above.

### Agrobacterium-mediated transient expression of CsGADs in Nicotiana benthamiana

*CsGADs* were subcloned into pCAMBIA3300-based binary vector with a S-tag at the N terminal. The constructed and blank vectors were transformed into *Agrobacterium* GV3101 by electroporation. The overnight *Agrobacterium* cultures were sedimented at 3500 × *g* for 15 min. and the pellets resuspended in a solution containing 10 mM MgCl_2_, 10 mM morpholineethanesulfonic acid (pH 5.6), and 100 μM acetosyringone to OD_600_ = 0.4^40^. Leaves of *N. benthamiana* were infiltrated by using a syringe without needle. After six days, GABA content in the leaves of *N. benthamiana* was determined by MSTFA derivatization and GC-MS analysis as described above.

### Statistical analysis

One way analysis of variance (ANOVA) was used to determine the differences among various treatments. A probability level of 5% (*p* ≤ 0.05) was considered as significant. The data were processed by using the SPSS statistical package (version 11.5).

## Additional Information

**How to cite this article**: Mei, X. *et al*. Dual mechanisms regulating *glutamate decarboxylases* and accumulation of gamma-aminobutyric acid in tea (*Camellia sinensis*) leaves exposed to multiple stresses. *Sci. Rep.* **6**, 23685; doi: 10.1038/srep23685 (2016).

## Supplementary Material

Supplementary Information
