# Supplementary material for: Dual mechanisms regulating glutamate decarboxylases and accumulation of gamma-aminobutyric acid in tea (Camellia sinensis) leaves exposed to multiple stresses
Source: Sci Rep. 2016 Mar 29;6:23685. doi: 10.1038/srep23685 (PMC4810369; doi:10.1038/srep23685)
Supplement: Supplementary Information [file srep23685-s1.pdf]

Title: Dual mechanisms regulating *glutamate decarboxylases* and accumulation of gamma-aminobutyric acid in tea (*Camellia sinensis*) leaves exposed to multiple stresses

Authors: Xin Mei [1,†], Yiyong Chen [1,2,†], Lingyun Zhang [3,†], Xiumin Fu [1], Qing Wei [1,3], Don Grierson [4], Ying Zhou [1], Yahui Huang [3], Fang Dong [5], Ziyin Yang [1,2*]

Affiliation:

[1] *Key Laboratory of South China Agricultural Plant Molecular Analysis and Genetic Improvement & Guangdong Provincial Key Laboratory of Applied Botany, South China Botanical Garden, Chinese Academy of Sciences, Xingke Road 723, Tianhe District, Guangzhou 510650, China*

[2] *University of Chinese Academy of Sciences, No.19A Yuquan Road, Beijing 100049, China*

[3] *College of Horticultural Science, South China Agricultural University, Wushan Road, Tianhe District, Guangzhou 510642, China*

[4] *Plant & Crop Sciences Division, School of Biosciences, University of Nottingham, Sutton Bonington Campus, Loughborough LE12 5RD, UK*

[5] *Guangdong Food and Drug Vocational College, Longdongbei Road 321, Tianhe District, Guangzhou 510520, China*

[*] Corresponding author. Ziyin Yang, Tel: +86-20-38072989; Email address: zyyang@scbg.ac.cn.

[†] These authors contributed equally to this work.

Supplementary Information

Table S1 Contents of free amino acids in picked (mechanically damaged) tea (*C. sinensis* cv. Jinxuan) leaves under aerobic and anoxic treatments.

Amino acid μg/g (fresh weight)	0 h	4 h		6 h	
		Aerobic	Anoxic	Aerobic	Anoxic
P-Ser	101.5±17.1b	66.5±4.5a	83.3±10.1ab	85.2±10.5ab	82.3±25.1ab
PEA	5.7±0.2a	8.3±2.2a	-	6.3±1.9a	-
Asp	122.0±14.7b	168.9±7.8c	13.5±2.3a	182.5±2.2c	12.3±0.9a
Thr	15.3±2.4a	23.5±0.9b	21.8±2.1ab	21.9±2.2ab	21.0±0.4b
Ser	38.3±6.9ab	40.8±2.7b	34.8±3.7ab	41.2±6.2ab	33.2±0.7a
Asn	5.8±0.4a	14.5±1.4c	9.1±1.2b	16.6±3.4c	9.7±0.8b
Glu	222.7±77.7c	335.7±20.1cd	101.9±17.6b	333.1±6.7d	45.8±10.5a
Thea	1075.9±122.8a	1276.6±112.5a	1281.9±187.2a	1202.3±385.2a	1136.5±216a
α-AAA	7.6±1.6a	8.9±1.1a	8.2±0.4a	9.2±2.9a	6.4±1.2a
Gly	8.6±0.2a	11.9±0.4b	18.3±2.8c	10.9±1.6ab	17.1±0.2c
Ala	8.6±1.2a	12.7±1.2b	169.6±14.9c	14.8±3.3ab	137.7±11.6c
Cit	4.5±0.3a	4.5±0.1a	-	4.1±0.5a	-
α-ABA	2.4±0.3a	2.6±0.1a	2.5±0.4a	2.7±0.4a	2.6±0.2a
Val	22.8±1.6b	3.5±0.8a	34.5±4.3c	14.3±18.9abc	2.0±0.1a
Cys	3.4±0.4b	3.1±0.3ab	2.8±0.4ab	2.2±0.7ab	2.0±0.4a
Ile	8.6±2.5a	15.8±0.9c	11.5±1.9ab	16.9±2.4c	12.5±0.6ab
Leu	5.2±1.3a	15.4±1.0b	13.8±1.6b	15.1±1.6b	13.9±0.5b
Tyr	18.0±2.9a	41.9±1.1b	56.0±6.2c	39.8±3.4b	57.2±2.9c
Phe	18.5±0.9b	36.5±1.3c	10.0±1.1a	42.6±6.1c	9.0±0.5a
β-Ala	2.2±0.2a	3.8±0.6b	7.7±0.9c	3.3±0.5ab	7.9±0.4c
β-ABA	0.6±0.1a	0.7±0.1a	0.6±0.2a	0.8±0.2a	0.7±0.3a
GABA	2.6±0.4a	2.0±0.5a	161.2±10.5b	4.3±1.3a	171.3±8.0b
Trp	16.7±4.2ab	22.9±1.5b	14.8±0.1a	21.5±5.9ab	16.9±3.9ab
$EOHNH_2$	22.1±1.8a	23.4±1.1a	22.4±4.1a	20.0±1.9a	22.5±0.7a
Lys	5.4±1.3a	11.1±1.1b	11.1±1.5b	10.0±1.1b	11.1±0.8b
1Mehis	6.2±0.3b	10.3±0.2c	3.8±0.3a	9.7±2.1bc	3.7±0.9a
His	3.3±1a	5.8±1.5ab	14.4±2.4c	5.8±0.6b	16.1±2.5c
Arg	9.8±6.7a	14.3±4.1a	16.2±5.7a	16.1±3.2a	11.6±2.6a

Data are expressed as mean ± S.D. (n=3). -, not detectable. P-Ser, Phosphoserine. PEA, Phosphorylethanolamine. Asp, Aspartic acid. Thr, Threonine. Ser, Serine. Asn, Asparagine. Glu, Glutamate. Thea, Theanine. α-AAA, α-amino acetic acid. Gly, Glycine. Ala, Alanine. Cit, Citrulline. α-ABA, α-Aminobutyric acid. Val, Valine. Cys, Cystine. Ile, Isoleucine. Leu, Leucine. Tyr, Tyrosine. Phe, Phenylalanine. β-Ala, β-Alanine. β-ABA, β-Aminobutyric acid. GABA, γ-Aminobutyric acid. Trp,

Tryptophan. $EOHNH_2$, Ethanolamine. Lys, Lysine. 1Mehis, 1-Methyl histidine. His, Histidine. Arg, Arginine. The test tea samples were prepared in June, 2013. Different means with different letters in the same row are significantly different from each other ($p \leq 0.05$).

Table S2 Primers used in this study

**For CsGAD CDS**	
CsGAD1F	5'-GCGGCCGCATGGTTCTGTCAAAGACAACATCG-3'
CsGAD1R	5'-GTCGACTTAGCAAACACCATTAGTCTTCTT-3'
CsGAD2F	5'-CCCGGATCCATGGTTCTCTCAAAGACTGCTTC-3'
CsGAD2R	5'-CCCAAGCTTCTAGCAAATCACTTGTGTTTTCC-3'
CsGAD3F	5'-ACGCGTCGACCCATGGTTATCTCAAATGTTCAAACC-3'
CsGAD3R	5'-ATAAGAATGCGGCCGCCTAACAAACTCCCTTTGTCTTACC-3'
**For mutants**	
CsGAD1ΔCR	5'-CCCAAGCTTTGACTCTTGGACTACAGC-3'
CsGAD2ΔCR	5'-CCCAAGCTTAGTCCCATTCTTCCCATTC-3'
CsGAD3ΔCR	5'-ATAAGAATGCGGCCGCCCTCTCTTCATCGCTT-3'
**For CsGAD transient expression**	
CsGAD1F	5'-CTAGTCTAGAATGCACCATCACCATCACCATCGCGTTCTGTCAAAGACAACA-3'
CsGAD1R	5'-CGCGGATCCCGTTAGCAAACACCATTAGTCT-3'
CsGAD2F	5'-CTAGTCTAGAATGCACCATCACCATCACCATCGCGTTCTCTCAAAGACTGC-3'
CsGAD2R	5'-CGCGGATCCCGCTAGCAAATCACTTGTGTT-3'
CsGAD3F	5'-CTAGTCTAGAATGCACCATCACCATCACCATCGCGTTATCTCAAATGTTCAAAC-3'
CsGAD3R	5'-CGCGTCGACCGCTAACAAACTCCCTTTGTCT-3'
**For qPCR**	
Csβ-ActinF	5'-GCCATATTTGATTGGAATGG-3'
Csβ-ActinR	5'-GGTGCCACAACCTTGATCTT-3'
CsGABA-T1F	5'-TTCACAGATAACAAGTCACCTAAT-3'
CsGABA-T1R	5'-TCTCACACTCTGCTCCAA-3'
CsGABA-T2F	5'-TAGTATGTTGGCACCATTCAC-3'
CsGABA-T2R	5'-ACCATAGACCAGCGAGAG-3'
CsGABA-T3F	5'-CGCAGTAGAAGTAGCAGTTG-3'
CsGABA-T3R	5'-GTCGGTTGTAAGAGATGTGAAT-3'
CsSSADH1F	5'-CCACCAAGTTCCAGAGATAC-3'
CsSSADH1R	5'-GCAAGTCCACAGGTAAGG-3'
CsSSADH2F	5'-ACATTCGCTATAACTTCACCAT-3'
CsSSADH2R	5'-TCCTCTCTCGGCAGATTAG-3'
CsGDH1F	5'-AGGTGGAGTTACGGTTAGTTA-3'
CsGDH1R	5'-GCACGAGCAACACGATTA-3'
CsGDH2F	5'-TTGTCCTCCTATTACCTCCAT-3'
CsGDH2R	5'-GAATCCAAGCCGAGAATGT-3'
CsSSR1F	5'-AACATCAGCAGAAGGACAT-3'
CsSSR1R	5'-ATCACCAAACACATCATATCAAG-3'
CsSSR2F	5'-GTGCCATTAGTTCGCCAAT-3'
CsSSR2R	5'-TGCTGATGCTTGAGAGGAA-3'
CsGAD1F	5'-AGTGACATCCAGAAAGTCTTGCT-3'
CsGAD1R	5'-CACCATTAGTCTTCTTCCTACTGAG-3'
CsGAD2F	5'-TTCGACATCTGCAAGGTGCTCCA-3'

CsGAD2R	5'-ACTTGTGTTTTCCTAGCCAAGAC-3'
CsGAD3F	5'-TTTCACATAACAAATGCAACGTC-3'
CsGAD3R	5'-CTCCCTTTGTCTTACCACCCATA-3'
AtACT2F	5'-TATCGCTGACCGTATGAGCA-3'
AtACT2R	5'-TTACCTGCTGGAATGTGCTG-3'
AtGAD1F	5'-CGCTGAGAGACTTGTGATCG-3'
AtGAD1R	5'-TTCTTCACCGTGACCATCAA-3'
AtGAD2F	5'-ACACTCGCGGAGAGACTTGT-3'
AtGAD2R	5'-CCATCAGAATCTCCTTCTCCA-3'
AtGAD3F	5'-GCCAAGATGGCTAGTGGAAA-3'
AtGAD3R	5'-GGTAATACTTGCTACTAACGGAACG-3'
AtGAD4F	5'-GTTCACGCCAAGATGGCTAA-3'
AtGAD4R	5'-GCAAATTGTGTTCTTGTTGGTC-3'
AtGAD5F	5'-CGAGGCCTTGCAGATAGACT-3'
AtGAD5R	5'-GGACATCTTGGCAGTCTTCAC-3'

GABA-T, GABA transaminase. GAD, glutamate decarboxylase. GDH, glutamate dehydrogenase. SSADH, succinic semialdehyde dehydrogenase. SSR, succinic semialdehyde reductase. The Accession Numbers of Csβ-Actin, CsGABA-T1, CsGABA-T2, CsSSADH1, CsSSADH2, CsGDH1, CsGDH2, CsSSR1, CsSSR2, and AtACT2 are HQ420251, GBBZ01005465, KA282884, HP727388, HP728333, GBBZ01002276, GBBZ01009549, GAAC01043686, GARM01012969, and NM_112764, respectively.

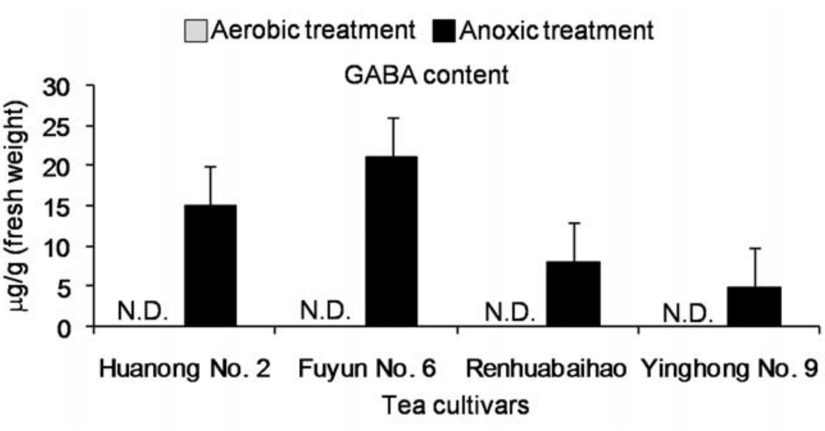

Figure S1 Contents of GABA in different cultivars of picked tea leaves (with mechanical damage) in air and anoxia.

GABA, gamma-aminobutyric acid. The tea leaves were treated under anoxic condition for 6 h. N.D., not detectable. Data are expressed as mean ± S.D. (n=3).

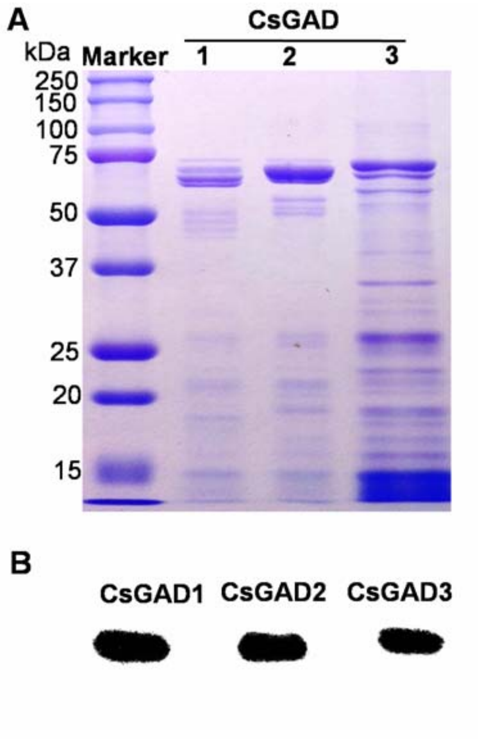

Figure S2 SDS-PAGE (A) and Western Blot (B) analyses of CsGADs expressed in *E. coli*. GAD, glutamate decarboxylase. The CsGADs were the partially purified expressed S-tagged recombinant proteins.

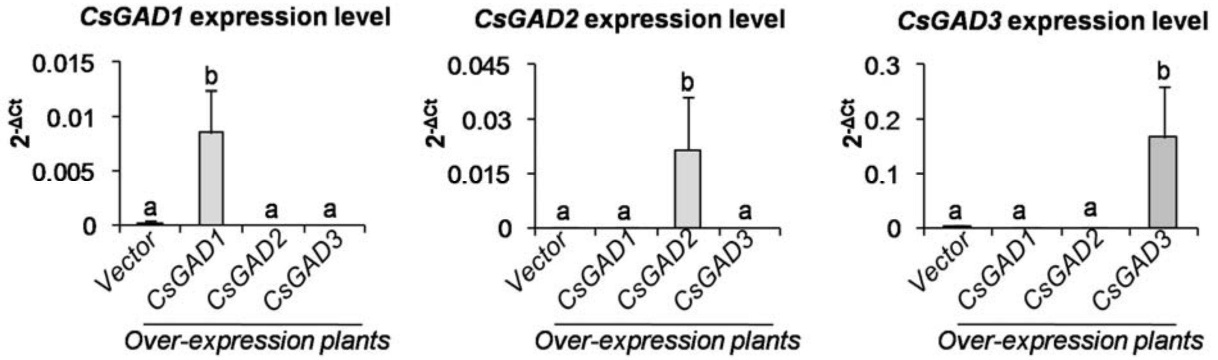

Figure S3 *Agrobacterium*-mediated transient expression of *CsGADs* mRNAs in *Nicotiana benthamiana*.

GAD, glutamate decarboxylase. $2^{-\Delta Ct}$ value was calculated based on comparison with beta-actin. Means distinguished with different letters are significantly different from each other ($p \leq 0.05$).

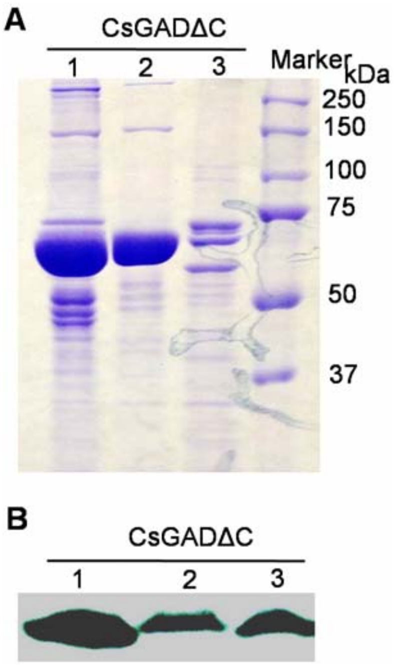

Figure S4 SDS-PAGE (A) and Western Blot (B) analyses of CsGADΔC s expressed in *E. coli.* GADΔC, C-terminal truncated glutamate decarboxylase. The loading amounts of CsGAD1ΔC, 2ΔC and 3ΔC were 0.3 μg, 0.1 μg, and 2 μg, respectively. The expressed CsGADΔCs S-tagged recombinant proteins, were detected using S-tag antibody.

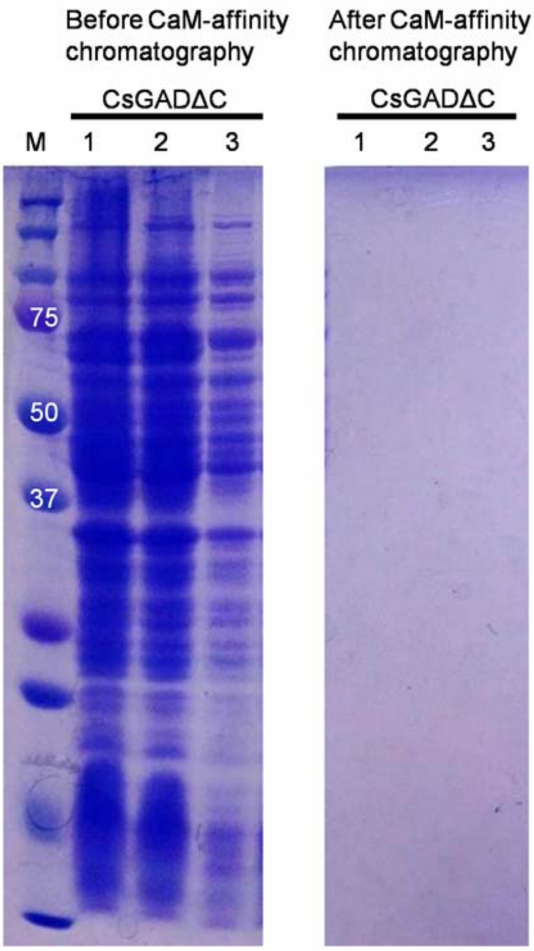

Figure S5 Determination of CaM-binding ability of CsGADΔCs expressed in *E. coli.*

GADΔC, C-terminal truncated glutamate decarboxylase. The CaM-affinity chromatography only detects proteins with CaM-binding ability. Any protein eluted from the CaM-affinity chromatography column is detected by SDS-PAGE, and is determined as having CaM-binding ability. No CsGADΔCs proteins were visible by SDS-PAGE, suggesting that the CsGADΔCs had no CaM-binding ability. M, molecular weight markers. A positive control was shown in the Figure 4A, suggesting that only the full-length CsGAD1 had CaM-binding ability.

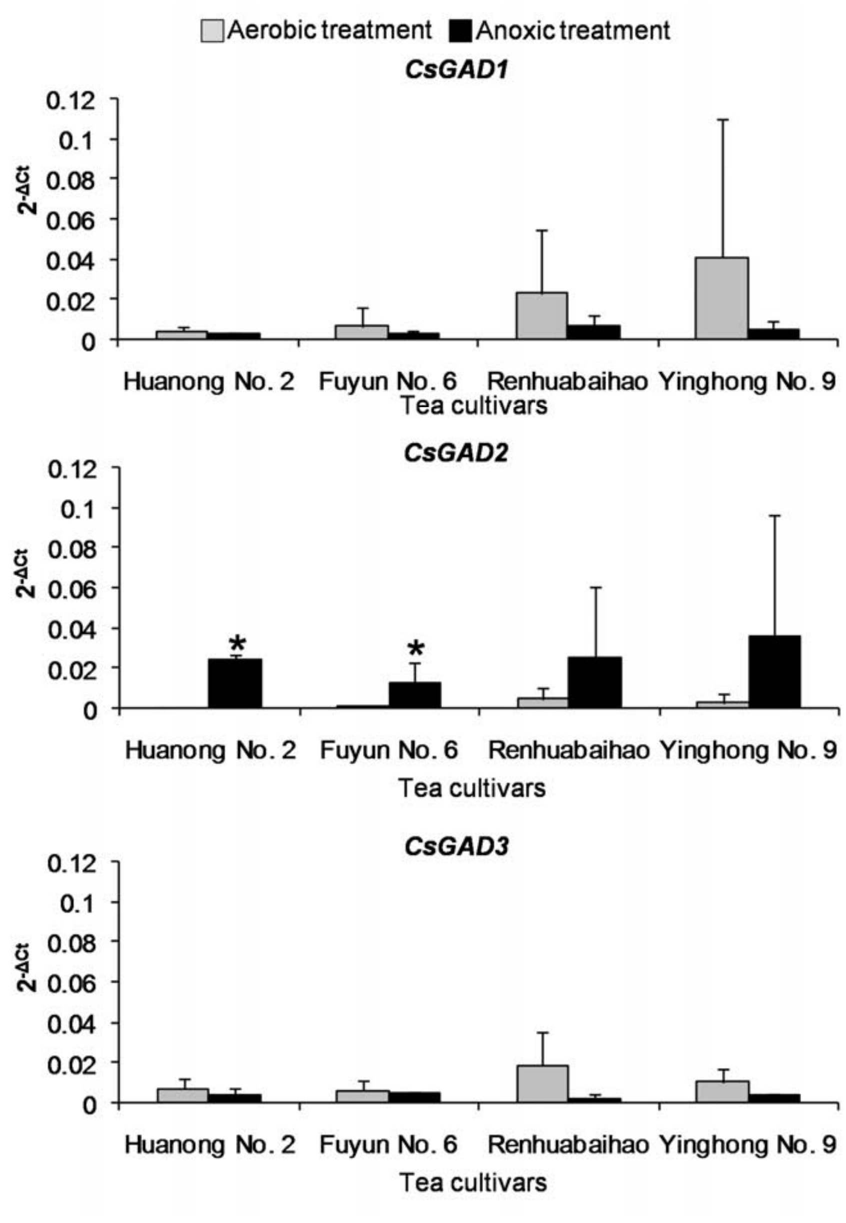

Figure S6 Expression levels of *CsGADs* in picked (mechanically damaged) tea leaves of different cultivars under aerobic and anoxic treatments

GAD, glutamate decarboxylase. The tea leaves were treated under anoxic condition for 6 h. Data are expressed as mean ± S.D. (n=3). $2^{-\Delta Ct}$ value was calculated based on comparison with beta-actin. *, $p < 0.05$.

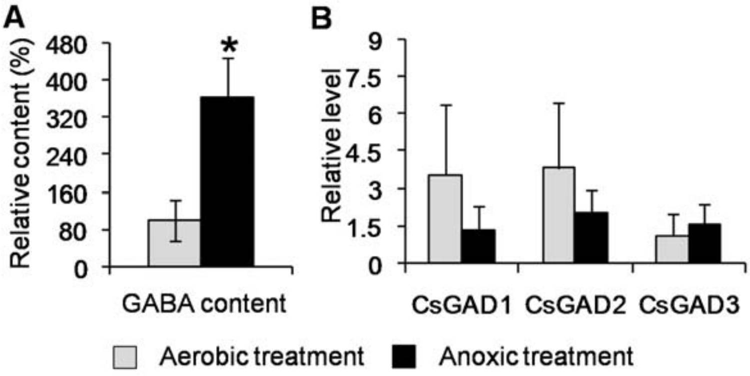

Figure S7 Effect of anoxia treatment on the GABA content and *CsGADs* mRNA levels in intact tea leaves

GABA, gamma-aminobutyric acid. GAD, glutamate decarboxylase. Tea leaves (*C. sinensis* cv. Jinxuan) were treated under anoxic conditions for 6 h. (A) The relative content (%) of GABA was calculated based on the GABA content of aerobic treatment (set as 100%). "*" indicates that anoxic treatment significantly elevated GABA content ($p < 0.05$). (B) Transcript abundance was calculated based on the difference in cycle threshold (Ct) values between *CsGADs* and beta-actin transcripts by the normalized relative quantification $2^{-\Delta\Delta Ct}$ method. The expression levels of the *CsGADs* in the tea leaves picked at 0 h were defined as 1.

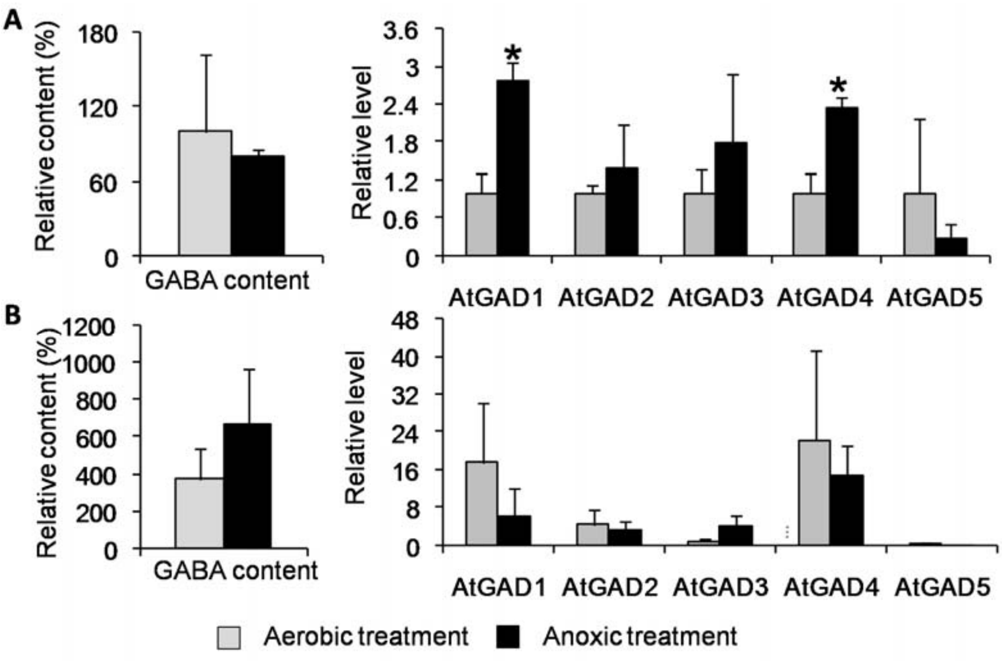

Figure S8 Effect of anoxia treatment on GABA content and levels of *AtGADs* mRNAs in the intact (A) and picked leaves (B) of *Arabidopsis thaliana*

GABA, gamma-aminobutyric acid. GAD, glutamate decarboxylase. Leaves were treated under conditions of anoxia for 6 h. The relative content (%) of GABA was calculated based on the GABA content of intact leaves under aerobic treatment (set as 100%). "*" indicates that anoxia significantly elevated *AtGAD1* and *AtGAD4* expression levels in the intact leaves ($p < 0.05$). Transcript abundance was calculated based on the difference in cycle threshold (Ct) values between *AtGADs* and beta-actin transcripts by the normalized relative quantification $2^{-\Delta\Delta Ct}$ method. The expression levels of the *AtGADs* of intact leaves under aerobic treatment were defined as 1.

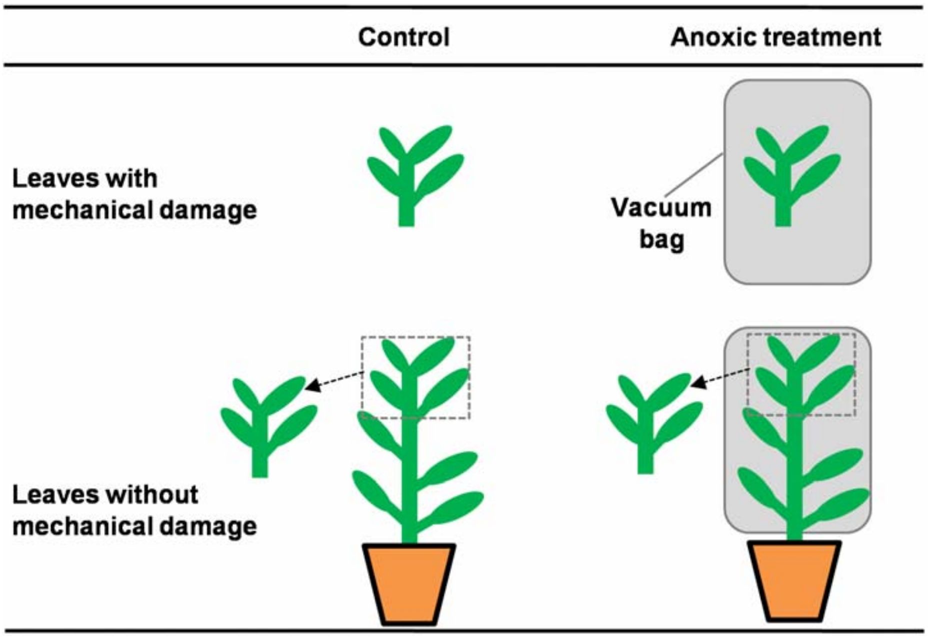

Figure S9 Anoxic treatments and mechanical damage applied to tea leaves.

Tea leaves (*C. sinensis* cv. "Jinxuan") were obtained from the tea seedlings grown under a controlled condition of 22-25°C and 12 h light/ 12 h dark. The anoxic treatments were carried out using a Vacuum Sealer (Deni, TVS-2013) at 25°C, 70% humidity in the dark. The air in the vacuum bag was drawn out by the Vacuum Sealer. The mechanical damage was induced by picking.
